# The Fate of Colour-Blind Sailors

**Published:** 1895-11-02

**Authors:** 


### THE FATE OF COLOUR-BLIND SAILORS.

MR. P. H. BICKERTON, ophthalmic surgeon to the Liverpool Royal Infirmary, brought what is nothing less than a public scandal before the annual meeting of the British Medical Association in July of the present year, a scandal of a kind involving many serious and dangerous consequences, one or two of which we desire to call attention to. Competent oculists have long been insisting that all persons connected with the navigation of ships, as well as all connected with the running of railway trains, should be men of perfect faculties as to eyesight, hearing, and so on. The reasons for this are so obvious that to argue the point would be ridiculous. As a matter of fact, nobody denies that sailors should be men of eyesight as perfect as is possible. But when it comes to applying such scientific and practical methods as will secure perfect eyesight in every boy or youth who proposes to follow the seafaring life, then difficulties of all kinds appear to spring up as if at the touch of a magic wand. In practice it comes to this: that anybody who pleases may commence the seafaring life, and he may continue it for as many years as he pleases if he be a person who has no desire to make the best of himself. But if, unfortunately for himself, he should develop a desire to improve his condition, he is obliged to submit his eyes for examination, and if he happen to be either colour blind or defective in sight, he is straightway told that he must leave the sea, or be content to be a plain A.B. to the end of the chapter.

Certain consequences spring from this condition of things which grieve the sympathetic and exasperate the practical. So much is this the case that if there were but any method of getting at all the sympathetic and all the practical members of the community in one supreme effort, there is no doubt that the new President of the Board of Trade would wish that his predecessors in office had shown a greater regard for practical science and a much diminished respect for permanent officialism and red-tape. Let us think for a moment of the intolerable hardships to which the best of our sailors are put who may find out when they are full-grown men that they are cursed with colour-blindness or defective sight. Of course, no boy ought to be apprenticed to a seafaring life without being first examined by an expert as to the condition of his eyesight. If he were so examined he could be informed, whilst still a boy, whether or not his eyesight were perfect, and whether or not he was fit for a seafaring life. In that case no harm would be done. He would simply devote himself to a different occupation in which the condition of the eyesight would be of less importance. But is not this expert examination of the eyesight of sailor apprentices already compulsory? it may be asked. By no means. Not only is it not compulsory, but the permanent officials at the Board of Trade seem to employ every means in their power to prevent its being made compulsory; and the late President of the Board, Mr. Bryce, appears to have taken a positive pleasure in upholding their wicked policy in every possible way. No doubt Mr. Bryce might have something to say in his own defence; but without waiting to consider that, let us lay to heart some of the practical results of this peculiar state of affairs. As we have said, there is no expert examination of boys as to their eyesight before apprenticeship to the sea, and, consequently, all our merchant sailors, through no fault of their own, grow to manhood in complete ignorance of their condition in this respect. Those of them who are smart and bright naturally want to advance themselves. So soon as they show any desire, after or during their apprenticeship, to aim at distinction in their calling, they are compulsorily subjected to an examination of the eyesight; and then, if any defect be found, comes the shattering of all their hopes and ambitions at a single blow. Mr. Bickerton told the British Medical Association of one young man, whose proper ambition to qualify himself for the position of mate having been thus rudely annihilated, straightway attempted suicide in consequence; and of another who, having become a captain without examination, had for some reason been examined, and having been found colour-blind, was dismissed his ship. The latter, not being able to obtain other employment, drooped and died within a twelvemonth. He told also of 165 other young men, who, after being allowed to complete their apprenticeship, had been found either colour-blind or defective in sight, and who, without any compensation whatever, were informed that they were disqualified for the sailor's life. All these circumstances are the record of less than a year's working of the compulsory eyesight examination system as now conducted.

Of course every scientific and every practical person recognizes that the eyesight of sailors should be examined, and that neither colour-blind nor near-sighted people should be employed at sea. The employment of such persons may indeed be the cause of some of the most tragic disasters of modern times. But a very simple method of procedure would both prevent the possibility of a certain class of disasters at sea, and also entirely obviate the ncessity for throwing some hundreds of deserving young men helpless upon the world every year. All that is required is that every youth who desires to follow the seafaring life shall be examined as to the condition of his eyes by a competent expert: examined, that is, before the commencement of his apprenticeship. By this simple means, as we have already said, a certain class of marine disasters would be entirely prevented, and such shameful injustice to young and deserving men as we have pointed out would be possible no longer. What hinders that this simple thing shall be done? Absolutely nothing but the wilful ignorance, or worse, of the permanent officials of the Board of Trade. No doubt this universal examination by experts would cost a little money every year—a mere trifle compared with the advantages to be gained. But is it right, is it English common-sense, that all travellers by sea should be needlessly exposed to manifold dangerous risks, and that shameful injustice should be done to a large class of deserving young men, merely because permanent officialism refuses to recognise an elementary scientific fact?

